# Laser-driven multi-MeV high-purity proton acceleration via anisotropic ambipolar expansion of micron-scale hydrogen clusters

**DOI:** 10.1038/s41598-022-18710-x

**Published:** 2022-10-12

**Authors:** Satoshi Jinno, Masato Kanasaki, Takafumi Asai, Ryutaro Matsui, Alexander S. Pirozhkov, Koichi Ogura, Akito Sagisaka, Yasuhiro Miyasaka, Nobuhiko Nakanii, Masaki Kando, Nobuko Kitagawa, Kunihiro Morishima, Satoshi Kodaira, Yasuaki Kishimoto, Tomoya Yamauchi, Mitsuru Uesaka, Hiromitsu Kiriyama, Yuji Fukuda

**Affiliations:** 1 Nuclear Professional School, School of Engineering, The University of Tokyo, 2-22 Shirakata Shirane, Tokai, Naka, Ibaraki 319-1188 Japan; 2 Present Address: Tono Geoscience Center, Japan Atomic Energy Agency (JAEA), 959-31, Jorinji, Izumi-cho, Toki, Gifu 509-5102 Japan; 3 Graduate School of Maritime Sciences, Kobe University, 5-1-1 Fukaeminamimachi, Higashinada, Kobe, Hyogo 658-0022 Japan; 4 Graduate School of Energy Science, Kyoto University, Gokasho, Uji, Kyoto 611-0011 Japan; 5 Kansai Photon Science Institute (KPSI), National Institutes for Quantum Science and Technology (QST), 8-1-7 Umemidai, Kizugawa, Kyoto 619-0215 Japan; 6 Nagoya University, Furo-cho, Nagoya, Aichi 464-8602 Japan; 7 National Institute of Radiological Sciences (NIRS), National Institutes for Quantum Science and Technology (QST), 4-9-1 Anagawa, Chiba, Chiba 263-8555 Japan; 8 Non-linear / Non-Equilibrium Plasma Science Research UNIT, Kyoto University, Gokasho, Uji, Kyoto, 611-0011 Japan; 9 Institute of Advanced Energy, Kyoto University, Gokasho, Uji, Kyoto, 611-0011 Japan

**Keywords:** Laser-produced plasmas, Plasma-based accelerators, High-field lasers, Ultrafast lasers, Nanoparticles

## Abstract

Multi-MeV high-purity proton acceleration by using a hydrogen cluster target irradiated with repetitive, relativistic intensity laser pulses has been demonstrated. Statistical analysis of hundreds of data sets highlights the existence of markedly high energy protons produced from the laser-irradiated clusters with micron-scale diameters. The spatial distribution of the accelerated protons is found to be anisotropic, where the higher energy protons are preferentially accelerated along the laser propagation direction due to the relativistic effect. These features are supported by three-dimensional (3D) particle-in-cell (PIC) simulations, which show that directional, higher energy protons are generated via the anisotropic ambipolar expansion of the micron-scale clusters. The number of protons accelerating along the laser propagation direction is found to be as high as 1.6 $\pm \,{0.3}$ $\times$ 10$^9$/MeV/sr/shot with an energy of 2.8 $\pm \,{1.9}$ MeV, indicating that laser-driven proton acceleration using the micron-scale hydrogen clusters is promising as a compact, repetitive, multi-MeV high-purity proton source for various applications.

## Introduction

The recent advancements in both laser^1^ and target fabrication techniques^2^ have led to enhancement of the laser-driven ion acceleration energies to close to 100 MeV/u^3–5^. The unique characteristics of ultrashort pulses and point sources possessed by laser-accelerated ions have potential applications for various research fields, such as proton radiography^6,7^, fast ignition^8,9^, nuclear science^10^, heavy particle radiotherapy^11^ and radiation-induced processes in matter^12,13^. In laser-driven ion acceleration experiments, thin films with thicknesses from nanometers to micrometers have been widely utilized as targets for laser irradiation^14,15^. However, in such experiments, regardless of the target material, not only protons, but also other ion species, such as carbon and oxygen, all of which originate from surface contaminants of thin films, are accelerated together, making the production of high-purity proton beams unrealistic.

From the viewpoint of practical applications, impurity-free multi-MeV proton beams have a great advantage. Therefore, laser acceleration of protons with various types of hydrogen targets has recently attracted much attention. For example, hydrogen droplets with diameters down to 10 $\upmu$m^16,17^, thin solid-hydrogen slabs^18^ and ribbons^19–22^ with thicknesses in the tens of $\upmu$m range, and liquid cryogenic microjets^23–26^ have been proposed and utilized in laser acceleration experiments. In addition, a high-density hydrogen gas jet has been utilized in laser acceleration experiments as a near critical density medium for near-infrared^27–29^ and infrared^30^ lasers.

Here, to increase the possibility of high-purity proton acceleration, we employ a different approach using micron-scale hydrogen clusters with a spherical shape^31,32^, which is an alternative to other types of hydrogen targets that allows very efficient coupling with laser pulses compared to planer-shaped targets^33^, thus exhibiting prominent linear and nonlinear dynamics and associated optical properties^34–36^ even in the radiation-dominant regime^37^. Notably, unlike previous approaches with the nanometer-scale hydrogen clusters^38–42^, this approach uses micron-scale hydrogen clusters, which exhibit a unique laser-cluster interaction. Satisfying certain conditions of laser properties and cluster size, the internal degree of freedom of micron-scale hydrogen clusters causing converging shock has been utilized in achieving quasimonoenergetic protons up to the sub-GeV energy range^43^.

In this paper, we report the experimental demonstration of laser-driven multi-MeV high-purity proton acceleration utilizing the micron-scale hydrogen clusters. Due to the replenishable target and the repetition rate laser, statistical analysis of hundreds of data sets has been conducted, which highlights the existence of markedly high energy protons generated from the laser-irradiated micron-scale clusters. Three-dimensional particle-in-cell (PIC) simulations suggest that the anisotropic ambipolar expansion mechanism works for the laser-irradiated micron-scale clusters, which gives multi-MeV protons of over 10 MeV in the laser propagation direction.

## Results

**Figure 1:**
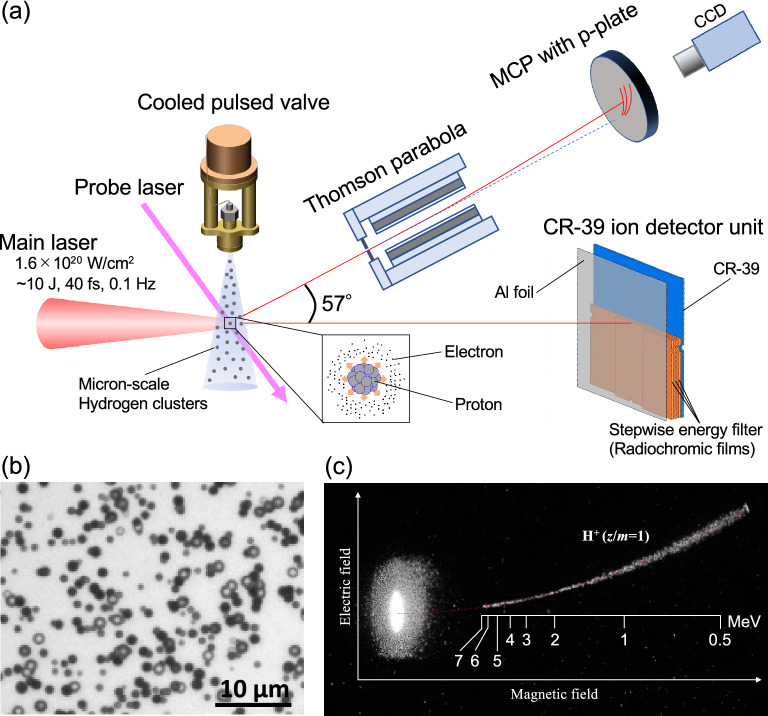
Experimental setup and proton signals. (**a**) Schematic of the experimental setup for laser-driven proton acceleration. (**b**) Micrograph of etch pits for > 0.93 MeV protons registered on the front surface of the CR-39 plate at the nozzle temperature of 25 K. (**c**) Single-shot proton signals detected using the real-time-type Thomson parabola at the nozzle temperature of 25 K. The vertically long elliptical bright spot is the laser focus area. The red dotted line represents the calculated ion trace with *z*/*m* = 1.

The experiment was conducted using the repetitive petawatt J-KAREN-P laser facility at QST-KPSI, which is based on an OPCPA/Ti:sapphire hybrid architecture^44,45^. A schematic of the experimental setup is shown in Fig. 1a. The laser delivers 37 $\pm \,{5}$ fs duration (FWHM) pulses of 9 J energy to the target at a 0.1 Hz repetition rate with a contrast ratio of 10$^{-11}$, which lasts until 200 ps before the main pulse^44^. The laser pulse was split into a main pulse and a lower energy probe pulse. The main laser pulse was focused to a 20 $\upmu$m diameter ($1/e^2$) spot using an F/10 off-axis parabolic mirror, which yielded a peak intensity of $1.6\times 10^{20}$ W/cm$^2$ under vacuum. High energy ions were generated when the laser pulses were focused into the center of the cluster gas flow at a distance of 15–20 mm from the nozzle outlet. The laser propagation in the cluster gas flow was monitored by a shadowgraph image with the probe laser, which orthogonally intersected with the main laser. The pulsed valve opening timing was synchronized to the laser pulse to optimize the laser-cluster interactions. To characterize accelerated ion energies, two different types of ion detectors, an integration-type CR-39 plate installed along the laser propagation direction^46^ and a real-time-type Thomson parabola^47^ installed at an angle of 57$^\circ$ with respect to the laser propagation direction, were employed.

In the present experiment, the micron-scale hydrogen clusters were produced via spallation of the liquid or supercritical fluid phase of hydrogen, namely, by expanding supercooled (25 or 50 K), high-pressure (6 MPa) molecular hydrogen gas into vacuum through a conical nozzle with an orifice diameter of 250 $\upmu$m and a conical angle of 40$^{\circ }$ connected to an Even-Lavie pulsed valve (EL-5-H-2019, Atad Digital Technology Trading and Marketing Ltd.)^32,48^. To cool the gas temperature, the pulsed valve was attached to a compact closed-cycle helium refrigerator (RDK-408E2, Sumitomo Heavy Industries, Ltd.). The temperature of the pulsed valve and the nozzle was monitored using a silicon diode temperature sensor (DT-670C-SD, Lake Shore Cryotronics, Inc.) and stabilized using two high-power thick-film resisters (RTO 50, Vishay Intertechnology, Inc.) connected to a temperature controller (Model 335, Lake Shore Cryotronics, Inc.).

The size distribution of the micron-scale hydrogen clusters was evaluated by Mie scattering measurements^31,32^. With a nozzle temperature of 50 K, the size distribution was mostly peaked at 0.3 $\upmu$m in diameter, while with a nozzle temperature of 25 K, in addition to the 0.3 $\upmu$m clusters, micron-scale hydrogen clusters were effectively generated, and the size of the micron-scale clusters was found to be widely distributed to up to 2.3 $\upmu$m in diameter^32^. Here, the mean cluster size was calculated to be 0.71 $\pm \,{0.04}\,\upmu$m and 0.33 $\pm \,{0.01}\,\upmu$m at nozzle temperatures of 25 and 50 K, respectively. The cluster density was estimated to be $2.0 \times 10^9$ and $4.9 \times 10^9$ clusters/cm$^3$ at nozzle temperatures of 25 and 50 K, respectively. The averaged intercluster distance was indirectly estimated to be 7.9 and 5.9 $\upmu$m at nozzle temperatures of 25 and 50 K, respectively, from the gas phase hydrogen density obtained by optical interferometry^32^.

## Discussion

Since the CR-39 detectors under the same etching condition have a potential to identify the ion species, especially between protons and other heavier ions^49^, protons on the CR-39 plate can be identified with the size of the etch pit opening. Figure 1b shows a micrograph of the etch pits that appeared on the CR-39 plate, where the sizes of all the etch pit openings (0.2–1.5 $\upmu$m$^2$) correspond to those of MeV-class protons obtained by the calibration study using conventional accelerators^46^. We could not find any etch pit whose size was larger than that of protons. Therefore, it is confirmed that all the accelerated ions belong to protons. In addition, as shown in Fig. 1c, only the single ion trace with $z/m=1$, i.e., protons, appeared on the fluorescent imaging plate of the Thomson parabola. These results clearly demonstrate that multi-MeV impurity-free proton acceleration was achieved via the laser-irradiated micron-scale hydrogen cluster target.

**Figure 2:**
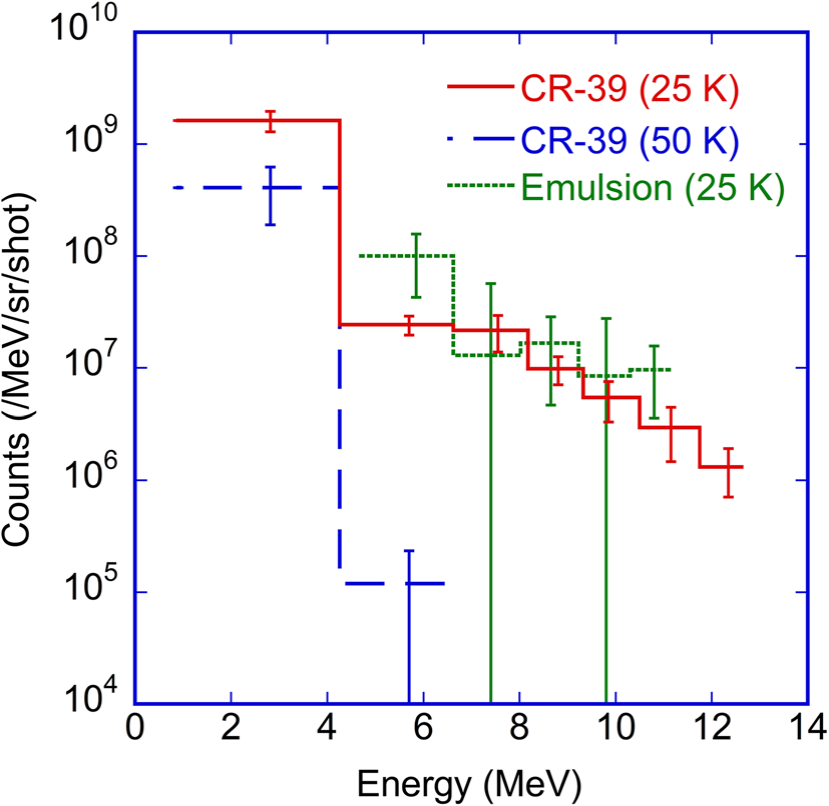
Energy spectra of laser-accelerated protons. The energy spectra measured using integration-type ion detector unit with the CR-39 plate at nozzle temperatures of 25 K (red solid line) and 50 K (blue broken line). The energy spectrum measured with the stack of nuclear emulsion films at the nozzle temperature of 25 K (green dotted line) is also shown as reference data^50^. The error bars on the ordinates represent the standard deviations of the number of the etch pit/track counts accumulated for over 70 laser shots, while those on the abscissa represent the measured energy range. These detector units were installed at a distance of 1.8 m from the laser focal point along the laser propagation direction.

Figure 2 shows the energy spectra of laser-accelerated protons measured using the 7 $\times$ 7 cm$^2$ integration-type detector unit with the CR-39 plate located along the laser propagation direction obtained at nozzle temperatures of 25 K and 50 K, where the ion signals were accumulated for 76 and 71 laser shots, respectively. For the case with a nozzle temperature of 25 K, the higher part of the energy spectrum obtained with the CR-39 plate is in good agreement with that obtained with the stack of nuclear emulsion films in the same series of experiments^50^, which confirms that the energy spectra measurements were conducted in a technically correct manner. The maximum energy of protons accelerating along the laser propagation direction is found to be 12.4 $\pm \,{0.6}$ MeV with the number of protons 1.3 $\pm \,{0.6}$ $\times$ 10$^6$/MeV/sr/shot, while the number of protons with the lower energy of 2.8 $\pm \,{1.9}$ MeV is found to be approximately 1.6 $\pm \,{0.3}$ $\times$ 10$^9$/MeV/sr/shot. The number of protons below 0.9 MeV detected by the CR-39 plate is estimated to be an order of magnitude larger than 10$^9$/MeV/sr/shot. For the case with the nozzle temperature of 50 K, the maximum proton energy of up to 5.7 $\pm \,{1.0}$ MeV is discernible.

**Figure 3:**
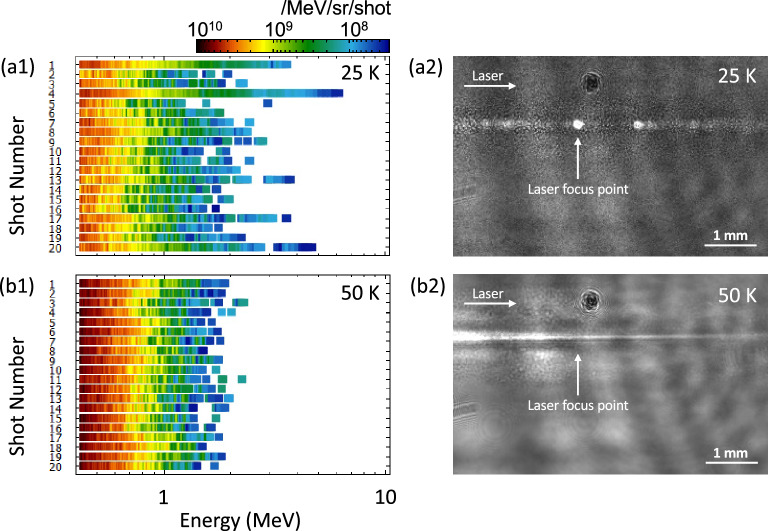
Shot-to-shot properties of laser-accelerated protons. Series of single-shot energy spectra for 20 consecutive shots measured using the real-time-type Thomson parabola ion detector at nozzle temperatures of (**a1**) 25 K and (**b1**) 50 K. The color scale displays the number of ions. The shadowgraph image of the laser propagation in the cluster gas flow with delay time at $\sim$ 100 ps at nozzle temperatures of (**a2**) 25 K and (**b2**) 50 K.

Figure 3a1, b1 show a series of single-shot energy spectra for 20 consecutive shots measured using the real-time-type Thomson parabola at nozzle temperatures of 25 K and 50 K, respectively. The maximum energies of accelerated protons in the energy spectra for 25 K have quite large fluctuations, while those for 50 K are stable. The production of lower energy protons ranging below $\sim$ 1 MeV is quite stable for both temperatures, and the number of such protons is found to be close to 10$^{10}$ /MeV/sr/shot. Note that the number of protons below $\sim$ 1 MeV measured by the real-time-type Thomson parabola is consistent with the value estimated by the CR-39 plate within the experimental error. The discernible fluctuations in the number of protons in the energy spectra for both temperatures come from the difference in the number of clusters inside the focal volume.

**Figure 4:**
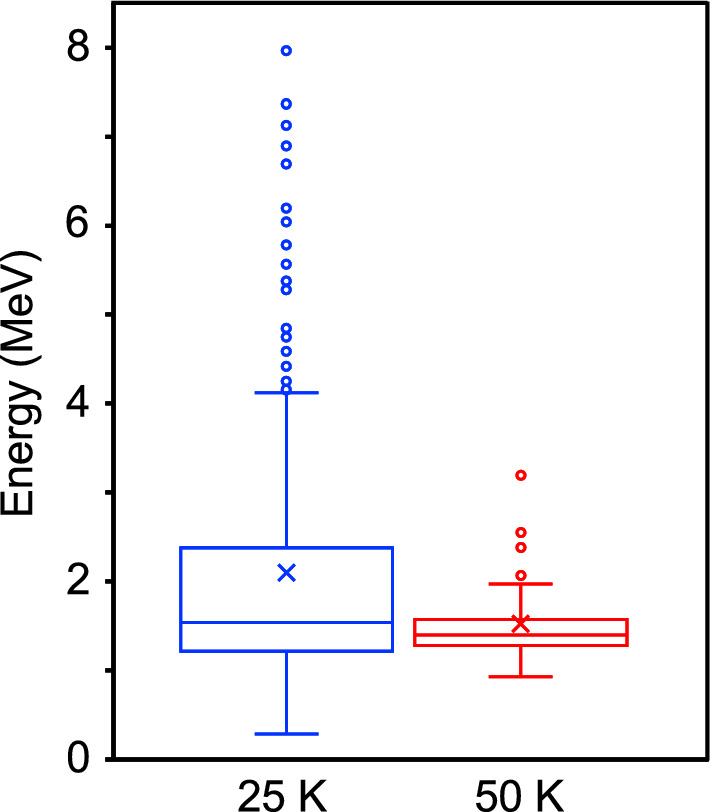
Statistical analysis highlights the existence of markedly high energy protons. Box-and-whisker plot for the maximum energies of the each data set for nozzle temperatures of 25 K (blue) and 50 K (red). The box is drawn from the 25th percentile (Q1) to the 75th percentile (Q3), with a horizontal line drawn in the middle to denote the median, and the cross mark represents the mean of the data set. The circles demonstrate outliers, which are defined as values greater than 1.5 IQR plus the third quartile.

Similar energy spectra data sets were obtained for 327 and 76 laser shots at nozzle temperatures of 25 K and 50 K, respectively. To understand the locality, spread, and skewness of the data sets, a box-and-whisker plot with whiskers drawn within the 1.5 interquartile range (IQR)^51^ was created for the maximum energies of each data set (see Fig. 4). Here, the box is drawn from the 25th percentile (Q1) to the 75th percentile (Q3) with a horizontal line drawn in the middle to denote the median, and the cross mark represents the mean of the data set. First, both data sets are clearly far from the normal distribution, especially in the case for the nozzle temperature of 25 K, and there exists strong inhomogeneity. Second, half of the data set is concentrated between 1.2 and 2.4 MeV for the nozzle temperature of 25 K, while it is concentrated in a much narrower region between 1.3 and 1.6 MeV for the nozzle temperature of 50 K. Third, the circles plotted on the box-plot demonstrate outliers, which are defined as values greater than 1.5 IQR plus the third quartile. The number of outliers observed for the nozzle temperature of 25 K indicates the strong inhomogeneity of the data set, while the outliers are less observable for the nozzle temperature of 50 K. This result can be explained in relation to the difference in the size distributions of the clusters with respect to the nozzle temperature: For the 50 K case, only clusters with a monolithic size distribution peaking at 0.33 $\pm \,{0.01}\,\upmu$m exist^32^, which results in Coulomb explosion of equivalent scale and gives almost the same energy spectra (see Fig. 3b1). In contrast, for the 25 K case, clusters with a widely spread size distribution exist^32^, where, in addition to the major component peaking at 0.33 $\pm \,{0.01}\,\upmu$m, a small number of larger sized clusters with diameters over 1 $\upmu$m exist, which occasionally produce higher energy tail protons if the laser pulse hits such micron-scale clusters. This hypothesis is supported by the shadowgraph images for the nozzle temperature of 25 K (see Fig. 3a2), which reveal that the higher energy tail protons are produced when the large bright spot image of self-emission from clusters appeared on the image, i.e. when the laser pulse hit micron-scale clusters, the large fluctuation in the energy spectra appeared. On the other hand, no bright spot was observed on the shadowgraph images for 50 K. The images were almost the same for every laser shot (see Fig. 3b2).

Comparing the maximum proton energies measured in the different directions at the nozzle temperature of 25 K, another interesting point is noted: The maximum proton energy of 12.4 MeV $\pm \,{0.6}$ MeV measured using the integration-type CR-39 plate in the laser propagation direction is found to be much larger than the value of 7.4 MeV $\pm \,{0.5}$ MeV measured using the real-time-type Thomson parabola at an angle of 57$^\circ$ with respect to the laser propagation direction, which indicates that the acceleration dynamics in the laser plasmas created via the laser-cluster interaction have inhomogeneity. At the present study, however, we are not able to discuss shot-to-shot properties of the accelerated proton spectrum in the laser propagation direction. The experiment using the 2nd real-time-type Thomson parabola in the laser propagation direction is now under way, whose results will be presented elsewhere in the near future.

**Figure 5:**
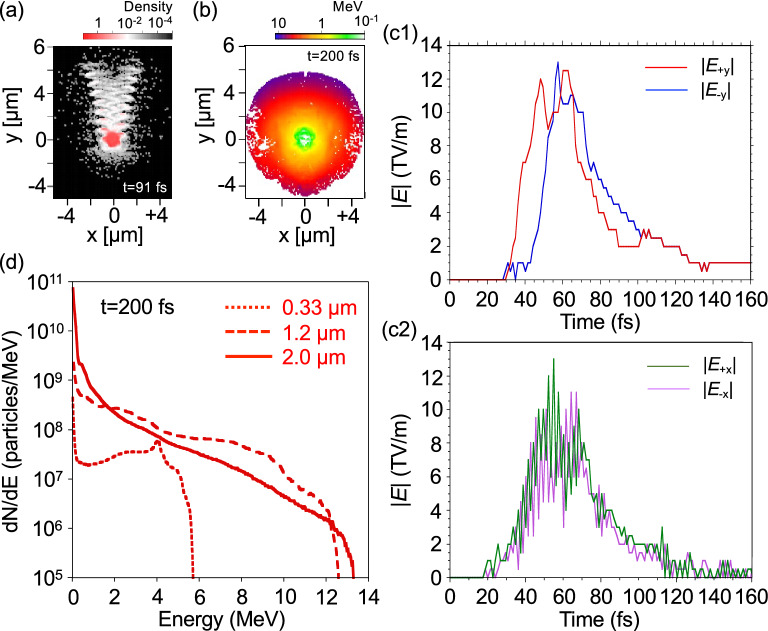
Anisotropic ambipolar expansion of the micron-scale hydrogen clusters. 2D images of (**a**) electron density and (**b**) ion kinetic energy distributions in the x–y plane for a hydrogen cluster with a diameter of 1.2 $\upmu$m at different times. The density is normalized by the initial charge density of the cluster electrons and displayed on a log scale. Temporal evolutions of electric field intensity at the exploding front of the cluster (**c1**) in the laser propagation direction and (**c2**) in the transverse direction for a hydrogen cluster with a diameter of 1.2 $\upmu$m. (**d**) Proton energy spectra for clusters with diameters of 0.33, 1.2, and 2.0 $\upmu$m. The proton number is evaluated using a real value by multiplying the particle weight by the PIC particle number.

To capture the essential dynamics of acceleration, the interaction processes of a single hydrogen cluster having a diameter of 0.33, 1.2, or 2.0 $\upmu$m with an intense laser pulse were investigated with the three-dimensional (3D) particle-in-cell (PIC) simulations (see Fig. 5). When the leading edge of the laser pulse reaches the cluster, the laser field expels electrons from the peripheral region of the cluster, and the expelled electrons are pushed forward along the general laser propagation direction, forming waves in the electron density due to the relativistic effect caused by the Lorentz force $v \times B$.

In the case of micron-scale clusters with diameters of 1.2 and 2.0 $\upmu$m, most of the electrons, 98.4% and 95.7%, respectively, remain unstripped inside of the clusters (see Fig. 5a). In this case, the ion dynamics are partially coupled to the electron dynamics and the anisotropic ambipolar expansion mechanism works for the laser-irradiated micron-scale clusters: The peripheral region of the cluster undergoes anisotropic Coulomb explosion that produces the largest energy ions along the laser propagation direction, while the core region of the cluster undergoes a hydrodynamic expansion that produces the smaller energy ions^32,35,43,52,53^. More specifically, Fig. 5c1 shows temporal evolutions of electric field intensity ${\vert }E{\vert }$ at the exploding front of the cluster in the laser propagation direction. At an early stage of the interaction (*t* = 30–50 fs), the electric field intensity in the laser propagation direction ${\vert }E_{+y}{\vert }$ rises up much earlier than that in the opposite direction ${\vert }E_{-y}{\vert }$, resulting in effective acceleration of protons in the laser propagation direction, i.e., the expelled electrons moving in the laser propagation direction drag protons located at the exploding front, producing an anisotropy in Coulomb explosion. On the other hand, a temporal evolution of oscillating electric field intensity ${\vert }E_{\pm {x}}{\vert }$ in a transverse direction produces no anisotropy (Fig. 5c2). Therefore, the resultant ambipolar expansion becomes anisotropic in the laser propagation direction, as shown in Fig. 5b, which supports the experimental observation. Another characteristic of ambipolar expansion is that the maximum ion energy does not sensitively depend on the size of clusters since only the peripheral region, the depth of which is determined by the electron skin depth, of the cluster undergoes Coulomb explosion. This trend is seen in Fig. 5d, which shows the maximum energies for the hydrogen clusters with diameters of 1.2 and 2.0 $\upmu$m are almost the same, 12.8 and 13.5 MeV, respectively. Note that according to the Coulomb explosion model, the maximum energies for the hydrogen clusters with diameters of 1.2 and 2.0 $\upmu$m are quite differently calculated as 100 and 276 MeV, respectively. Therefore, the anisotropic ambipolar expansion of clusters with diameters of 1.2 and 2.0 $\upmu$m corresponds well to the experimental observation of a maximum proton energy of 12.4 $\pm \,{0.6}$ MeV measured in the laser propagation direction with a nozzle temperature of 25 K including micron-scale hydrogen clusters.

In contrast, in the case of the cluster with a diameter of 0.33 $\upmu$m, only 0.57% of the electrons remain unstripped when the peak of the laser pulse reaches the cluster center. Therefore, the ion dynamics are practically decoupled from the electron dynamics, and the whole cluster is strongly positively charged, resulting in Coulomb explosion. In the case of Coulomb explosion^35^, the maximum energy for hydrogen clusters with diameters of 0.33 $\upmu$m is calculated as 7.5 MeV. In the present 3D PIC simulation, this results in a maximum proton energy of 5.8 MeV, as shown in Fig. 5d, which also corresponds well to the experimental observation of a maximum proton energy of 5.7 $\pm \,{1.0}$ MeV measured in the laser propagation direction at a nozzle temperature of 50 K (see Fig. 2).

## Conclusion

Multi-MeV protons are accelerated via laser irradiation of micron-scale hydrogen cluster targets at a laser intensity of $1.6 \times 10^{20}$ W/cm$^2$ with a 0.1 Hz repetition rate . The number of multi-MeV protons accelerating along the laser propagation direction is found to be much larger than 10$^{9}$/MeV/sr/shot. According to the statistical data analysis, markedly high-energy protons are generated from the micron-scale hydrogen clusters. The spatial distribution of the accelerated protons, in which the higher energy protons are preferentially accelerated along the laser propagation direction, can be attributed to the anisotropic ambipolar expansion of the micron-scale hydrogen clusters. The 3D PIC simulations with parameters that match the experimental conditions support these experimental observations. Inherently robust nature of anisotropic ambipolar expansion of the micron-scale hydrogen clusters is promising as a compact, repetitive, multi-MeV high-purity proton source for various applications. As a future prospect of laser-driven high-purity proton acceleration, the 3D PIC simulation predicts the generation of directed proton beams as high as 117 MeV via the anisotropic Coulomb explosion of the 1.2 $\upmu$m diameter hydrogen cluster when irradiated by a PW-class laser pulse with a peak intensity of $1\times 10^{22}$ W/cm$^2$^32,43^. For interaction of the PW-class laser pulses with the hydrogen clusters having the diameters larger than 1.6 $\upmu$m, a new scheme for achieving quasimonoenergetic proton bunch acceleration in excess of 300 MeV has been proposed, where the collisionless shock dynamics inside the micron-scale cluster coupled with relativistically induced transparency (RIT) effect of high-intensity laser plays an important role^43^.

## Methods

### Integration-type ion detector unit with the CR-39 plate

The energies of protons accelerated along the laser propagation direction were measured by using an integration-type detector unit composed of a 13 $\upmu$m thick aluminium foil, a stepwise energy filter consisting of three different sizes of radiochromic films (XR-RV3, Gafchromic), and a 7 $\times$ 7 cm$^2$ CR-39 (HAZLAS (TD-1), Fukuvi Chemical Industry) plate, which made it possible to construct an energy spectrum of the accelerated protons for an energy range from 930 keV to 20 MeV^46^. The detector unit was installed in the vacuum chamber at a distance of 1.8 m from the focal point along the laser propagation axis.

After the measurements, the CR-39 plate was chemically etched in stirred 6 M-KOH solution kept at 70 $^\circ$C for 30 min. By counting the number of etch pits on the CR-39 plate using a fast automated digital imaging optical microscope (HSP-1000, Seiko Precision Inc.), the energy spectrum was reconstructed by plotting the number of etch pits in each energy region defined by the stepwise energy moderator, the details of which are described in ref. [46].

### Real-time-type Thomson parabola ion detector

To understand the shot-to-shot properties of the accelerated proton beam, the accelerated proton energies were measured on a single shot basis using a real-time-type Thomson parabola spectrometer^47^, installed at an angle of 57$^\circ$ with respect to the laser propagation direction. In the real-time Thomson parabola, after passing through a 600 $\upmu$m-diameter entrance pinhole located 1.4 m from the laser focal point, ions travel through 100-mm homogeneous electric (400 V/mm) and magnetic (1.6 T) fields, where ions are differentiated by the charge-to-mass ratio and the energy, and are detected by a chevron microchannel plate (MCP) with a diameter of 75 mm equipped with a fluorescent (P20) imaging plate (3075PS, BURLE), where ions with the same charge-to-mass ratio trace the same parabola in the detector plane. The 2D image of the ion trace that appeared on the fluorescent imaging plate was monitored by the a 10-bit CMOS camera (DMK33GP1300, Imaging Source). From the brightness of the fluorescent imaging plate, the number of protons (/MeV/sr/shot) was roughly estimated using a calibration factor of the order of 10 counts/proton, obtained in a separate calibration experiment, where the solid angle is defined by the pinhole located at the entrance of the Thomson parabola spectrometer.

### Particle-in-cell (PIC) simulations

The 3D PIC simulations were conducted by employing the EPIC3D code^54^. A single hydrogen cluster with an electron density of $4.6 \times 10^{22}$ cm$^{-3}$(= 26.8 $n_c$) was placed at the center of a system with a size of $L_x$ = $L_z$ = 10.24 $\upmu$m and $L_y$ = 20.48 $\upmu$m; here, $n_c$ is the critical density of plasma corresponding to the laser wavelength $\lambda$ = 810 nm. The cluster was surrounded by ambient hydrogen gas with an electron density of $4.0 \times 10^{19}$ cm$^{-3}$ (= 0.024 $n_c$). A grid size of 80 nm was employed to resolve the cluster explosion dynamics. A plane-wave laser pulse linearly polarized in the *x* direction with a wavelength $\lambda$ = 810 nm and a pulse duration of 33 fs (FWHM) was propagated in the $+y$ direction. The peak intensity of the laser pulse was set to $1.2 \times 10^{20}$ W/cm$^2$ (corresponding to the normalized amplitude of a laser $a_0$ = 7.8, where $a_0$ = $eE/{m_e}c{\omega }$). The transparent boundary condition was used in the *y* direction, while the periodic boundary condition was used in the *x* and *z* directions. A fully ionized hydrogen plasma was employed as an initial condition. Note that the grid size is larger than the skin depth ${\delta }_{e}\sim 60$ nm of the present laser field including relativistic effect (the Lorentz factor $\gamma \sim 5.6$, where $\gamma$ = $\sqrt{1+{a_0^2}/2}$ for the linearly polarized laser pulse). By conducting 2D and 3D trial simulations with smaller grid sizes, it is confirmed that the present 3D simulations capture the essential dynamics inside the skin depth: They overestimate the laser absorption rate by $\sim$ 2.7% and the resultant maximum ion energy by $\sim$ 2.5%.

## Data Availability

The datasets used and/or analysed during the current study available from the corresponding author on reasonable request.
